# Biological and microbiological interactions of Ti-35Nb-7Zr alloy and its basic elements on bone marrow stromal cells: good prospects for bone tissue engineering

**DOI:** 10.1186/s40729-020-00261-3

**Published:** 2020-10-25

**Authors:** Daphne de Camargo Reis Mello, Lais Morandini Rodrigues, Fabia Zampieri D’Antola Mello, Thais Fernanda Gonçalves, Bento Ferreira, Sandra Giacomin Schneider, Luciane Dias de Oliveira, Luana Marotta Reis de Vasconcellos

**Affiliations:** 1 Department of Bioscience and Oral Diagnosis, São José dos Campos School of Dentistry, Universidade Estadual Paulista (UNESP), Av. Engenheiro Francisco José Longo, 777, São José dos Campos, SP 12245-000 Brazil; 2 Oakland University, Mathematics and Science, 318 Meadow Brook Rd, Rochester Hills, USA; 3 Escola de Engenharia de Lorena (EEL-USP), Pólo-Urbo Industrial, Gleba Al-6, S/N, Lorena, SP Brazil

**Keywords:** Ti-35Nb-7Zr alloy, Biofilm, Bone marrow cells, Osteogenesis

## Abstract

**Background:**

An effective biomaterial for bone replacement should have properties to avoid bacterial contamination and promote bone formation while inducing rapid cell differentiation simultaneously. Bone marrow stem cells are currently being investigated because of their known potential for differentiation in osteoblast lineage. This makes these cells a good option for stem cell-based therapy. We have aimed to analyze, in vitro, the potential of pure titanium (Ti), Ti-35Nb-7Zr alloy (A), niobium (Nb), and zirconia (Zr) to avoid the microorganisms *S. aureus* (S.a) and *P. aeruginosa* (P.a). Furthermore, our objective was to evaluate if the basic elements of Ti-35Nb-7Zr alloy have any influence on bone marrow stromal cells, the source of stem cells, and observe if these metals have properties to induce cell differentiation into osteoblasts.

**Methods:**

Bone marrow stromal cells (BMSC) were obtained from mice femurs and cultured in osteogenic media without dexamethasone as an external source of cell differentiation. The samples were divided into Ti-35Nb-7Zr alloy (A), pure titanium (Ti), Nb (niobium), and Zr (zirconia) and were characterized by scanning electron microscopy (SEM) and energy-dispersive X-ray spectroscopy (EDS). After predetermined periods, cell interaction, cytotoxicity, proliferation, and cell differentiation tests were performed. For monotypic biofilm formation, standardized suspensions (10^6^ cells/ml) with the microorganisms *S. aureus* (S.a) and *P. aeruginosa* (P.a) were cultured for 24 h on the samples and submitted to an MTT test.

**Results:**

All samples presented cell proliferation, growth, and spreading. All groups presented cell viability above 70%, but the alloy (A) showed better results, with statistical differences from Nb and Zr samples. Zr expressed higher ALP activity and was statistically different from the other groups (*p* < 0.05). In contrast, no statistical difference was observed between the samples as regards mineralization nodules. Lower biofilm formation of S.a and P.a. was observed on the Nb samples, with statistical differences from the other samples.

**Conclusion:**

Our results suggest that the basic elements present in the alloy have osteoinductive characteristics, and Zr has a good influence on bone marrow stromal cell differentiation. We also believe that Nb has the best potential for reducing the formation of microbial biofilms.

## Background

One of the most important discoveries of twentieth century dentistry was the process of osseointegration [1]. Implants are intended to remain in the human body for a long time and bear the same loads as the surrounding bone. Therefore, it is important that they not only allow osseointegration, but also present mechanical characteristics like bone tissue [2]. Titanium and its alloys are the most used metals in the biomedical field, because of their mechanical and biological properties [3]. One of the main alloys used is Ti-6Al-4V (titanium-6aluminum-4vanadium), but it has been reported that this alloy can release the aluminum (Al) element and can become toxic in the organism, besides having a greater modulus of elasticity (110 GPA) than cortical bone (30 GPA), resulting in stress shielding and consequent failure of the implant [4]. To avoid this phenomenon, an alternative is to use non-toxic neutral elements, including niobium (Nb) and zirconia (Zr), since both metals can be incorporated to pure titanium (Ti) and do not show cytotoxicity [5, 6]. One of the first ternary alloys with Ti, developed with low modulus, was Ti-13Nb-13Zr alloy. This alloy presents a mechanical strength, high corrosion resistance, and lower modulus of elasticity than Ti-6Al-4V and Ti-6Al-7Nb alloys, besides showing biocompatibility and osteoinduction characteristics [7–9].

Although the presence of non-toxic elements and a low modulus of elasticity is an important criterion for the development of implants, there are other issues involved in the manufacture of these biomaterials, mainly because it is known that the chemical composition of the different substrates used to produce different implants may directly affect microorganism adhesion and oral biofilm maturity [10, 11]. Two important bacterial strains, which have not been explored in studies involving titanium alloys are *Staphylococcus aureus* and *Pseudomonas aeruginosa.* There are few studies regarding these opportunistic pathogens, because the major focus has been on anaerobic gram-negative bacteria, which have been directly responsible for peri-implantitis [12]. However, some studies have shown that these bacteria can successfully attach to titanium surfaces [13–16] and can cause orthopedic device related infections, such as early mandibular osteomyelitis after implant surgery [17].

Bone marrow-derived stromal cells (BMSCs) have been the focus of some studies, mainly because they can be easily obtained; they are a good source of stem cells, with the potential to differentiate into osteoblasts and have strong proliferative capacity, being considered good candidates for bone tissue engineering [18]. He et al. [19] affirm that bone marrow stromal cells (BMSCs) are a source of progenitors of osteoblasts, are responsible for bone formation, and have a key role during bone repair. Therefore, our study was conducted to investigate if Ti-35Nb-7Zr and its basic elements—titanium, niobium, and zirconia—have the potential to decrease or avoid contamination by *Staphylococcus aureus* and *Pseudomonas aeruginosa* on their surfaces. Furthermore, we analyzed the osteoinductive properties of these metals, demonstrating that they can influence bone marrow stromal cells to differentiate in mature osteoblasts that can secret bone matrix proteins. In this study, we were able to show the capacity of these cells in proliferation and differentiation into osteoblasts, proving that this could be a good option for bone therapy in the future. We used media without an external inductive source—dexamethasone—which is an anti-inflammatory widely used as an in vitro supplement to promote osteogenic differentiation. However, in vivo studies show that the use of this anti-inflammatory can inhibit bone formation and induce bone resorption [20, 21].

## Materials and methods

### Sample fabrication and characterization

The Nb samples and the Ti-35Nb-7Zr alloy were produced at the Lorena School of Engineering, University of São Paulo (USP), in the Department of Materials Engineering. The pure elements for the preparation of the samples of Ti and Zr were acquired in the commercial market from Müller Metals Ind. Com. Ltda. To obtain the Ti-35Nb-7Zr alloy, the Ti, Nb, and Zr plates were cast in an arc melt furnace. All samples, regardless of material, were subjected to three ultrasonic washes. The samples were subdivided into groups according to the material: Ti-35Nb-7Zr alloy (A), Ti pure (Ti), niobium (Nb), and zirconia (Zr). All samples were cut 4 mm long, 4 mm wide, and 1.5 mm thick. Five samples per group were used for cellular tests (total of 40 samples) and 8 samples per group for tests with monotypic microbiological biofilms (total of 64 samples). The following images show the characterization of the samples that was performed by scanning electron microscopy (SEM) (Zeiss-EVO MA10, São Paulo, Brazil), and with a spectrophotometer with energy dispersion (EDS). The equipment operates in a high vacuum with a secondary electron detector at a voltage of 12.5 HV and 4.5 points.

### Isolation and culture of BMSCs

All animal procedures were performed according to the guidelines of the Research Ethics Committee of the School of Dentistry of São José dos Campos (protocol 006/2016-CEUA-ICT-UNESP). Twenty-three male Wistar-strain rats each with an average body weight of 250 g and 3 months of age were used from the vivarium of the UNESP Institute of Science and Technology of São José dos Campos. The animals were housed in individual cages, with freely available water and food, and an artificial day/night cycle of 12 h/12 h in an air-conditioned room. Euthanasia was performed by anesthetic overdose (ketamine and xylazine). The femurs were removed and placed in a carrier medium solution composed of minimal alpha MEM (Gibco) essential culture medium and gentamicin (500 μg/ml) (Gibco). Bone marrow cells were isolated by femur irrigation with supplemented total medium composed of minimal alpha MEM (Gibco) essential culture medium supplemented with 10% bovine fetal serum (SBF ) (LGC, Cotia, Sao Paulo, Brazil) and gentamicin (500 μg / ml) (Gibco) and inserted into 50-ml falcon-like tubes (TPP, Switzerland), which were then transferred to a 250-ml, 75-cm^2^ cell culture bottle (TPP, Switzerland) and were incubated in a stove at 37 °C at atmospheric humidity containing 5% CO_2_. The cells were selected for adhesion to polystyrene and expanded until the cells reached confluence characterized by the occupancy of more than 80% of the vial. All samples were autoclaved at 134 °C and, after the appropriate period, cell plating was performed. Cells were plated at 20,000 cells/well in 24-well plates. Osteogenic culture medium was made with alpha MEM (Gibco) essential culture medium supplemented as above described, and 5 mg/ml ascorbic acid (Neon) and 2.16 g of beta-glycerophosphate (Sigma-Aldrich) were added. During this procedure, the cells were incubated at 37 °C with atmospheric moisture containing 5% CO_2_. Cell development was assessed by reverse phase microscopy (Microscope Carl Zeiss Microimaging GmbH—Axiovert 40C, Germany). Five samples from each group were used, except for the cell adhesion test, and all assays were done in duplicates. The cellular response was investigated further by means of the proposed tests.

### Scanning electron microscope

Cell adhesion, morphology, and spreading on the surfaces were evaluated by FE-SEM (Zeiss-EVO MA10, São Paulo, Brazil) after 7 days of culture (*n* = 2 for each experimental group). The samples were chemically fixed with 4% paraformaldehyde and then dehydrated with ethanol, and before analysis, the samples were coated with a thin layer of gold using a sputter-coating system.

### Cell viability

After 7 days of cell culture, to quantify total viable bacterial cells adhered to the samples, MTT (3-(4,5-dimethylthiazol-2-yl)-2,5-diphenyltetrazolium bromide) was performed. In a 24-well plate, 0.5 mg/ml MTT was added in each well. The samples were incubated in a stove with 5% CO_2_ at 37 °C for 1 h. Dimethyl sulfoxide (DMSO, Sigma-Aldrich, St. Louis, MO) was then added to dissolve the formazan purple crystals of MTT. The data was measured in absorbance in the spectrophotometer (Micronal AJX 1900) at wavelength 570 nm.

### Cell proliferation and adhesion

To assess cell adhesion and proliferation ability, the bone marrow cells were cultured in the samples for 24 h. After this time, the culture medium was removed, and the samples were washed and fixed with 4% paraformaldehyde. The samples were washed again with PBS, and Dapi dye was added. The samples remained incubated at room temperature. Subsequently, 10 fields from each group were randomly captured using the Axio HBO 100 (Zeiss) microscope using a × 20 lens and the nuclei present in the samples were quantified using Image J® version 6.0 software (National Institutes of Health, Bethesda, MD, USA).

### Cell differentiation: osteogenesis

The total protein content was determined according to the method of Lowry et al. [22] using cells cultured for 10 days. The procedure was performed and cells in culture were placed in contact with the detergent sodium lauryl sulfate, and after cell lysis, were added to the Lowry reagent and subsequently to the folin. The absorbance was then measured at 680 nm in a spectrophotometer (Micronal AJX 1900). The total protein content was calculated from a standard curve determined from bovine albumin and expressed as μmolg/ml. Alkaline phosphatase activity was determined after 10 days of cell culture. A commercial kit (Labtest Diagnóstica, Belo Horizonte, BR) was used and the manufacturer’s instructions were followed. The absorbance was measured on a spectrophotometer (Micronal AJX 1900) using a wavelength of 590 nm and alkaline phosphatase activity was calculated from a standard curve using thymolphthalein on a scale of 0.012 to 0.4 μmol of thymolphthalein protein/hour/microgram. The formation of mineralization nodules was evaluated after 14 days of cell culture. The nodules were fixed with 2% Alizarin Red dye pH 4.2. Subsequently, the quantification of mineralized formations was performed, and the nodules were extracted with 10% acetic acid and then 10% ammonia hydroxide to neutralize the acid. The reading was performed on a microplate reader 59 (Biotek—EL808IU, USA), at a wavelength of 405 nm.

### Evaluation of the formation of microbial biofilms

The strains *Staphylococcus aureus* (ATCC 6538) and *Pseudomonas aeruginosa* (ATCC 15442) from the Laboratory of Microbiology and Immunology of UNESP were cultured on solid medium in BHI agar (Brain Heart Infusion—BHI for bacteria and yeast) and later in liquid medium (BHI) for 24 h in a stove at 37 °C. Then, each culture broth was standardized at 10^6^ cells/ml in a spectrophotometer (Micronal B-582, São Paulo, Brazil), and the sterilized samples of the Ti-35Nb-7Zr alloy and its basic elements were dispensed into 24-well plates (*n* = 8 for each experimental group) with 500 μl of artificial saliva on the orbital shaker. Afterwards the artificial saliva was removed and 1 ml BHI broth well, 1 ml of artificial saliva, and 100 μl of standardized microbial suspension were added to form monotypic biofilms of the selected microorganisms. After 24 h of incubation in the stove (37 °C), the samples were washed with a sterile physiological solution and 300 μl of MTT [3-4,5-dimethylthiazole] (Sigma-Aldrich) remaining in contact with the samples for 1 h incubation in a stove at 37 °C (5% CO_2_ for *S. mutans*) under light protection. This solution was then removed and 300 μl of dimethylsulfoxide (DMSO—Sigma-Aldrich) remained in the wells under incubation in a stove at 37 °C. Subsequently, the plate was stirred on an orbital table. One hundred microliters aliquots from each well were transferred to a 96-well plate (Greiner) and absorbance was measured on a spectrophotometer (Biotech EL808IU) using a wavelength of 570 nm.

### Statistical analysis

The data were analyzed by one-way ANOVA and if the differences were significant (*P* < 0.05), then analyzed by a multiple comparison test (Tukey). All statistical analyses were performed using GraphPad Prism software, version 6.00 (Graphpad Prism, Inc., San Diego, CA)

## Results

### Sample characterization

**Fig. 1:**
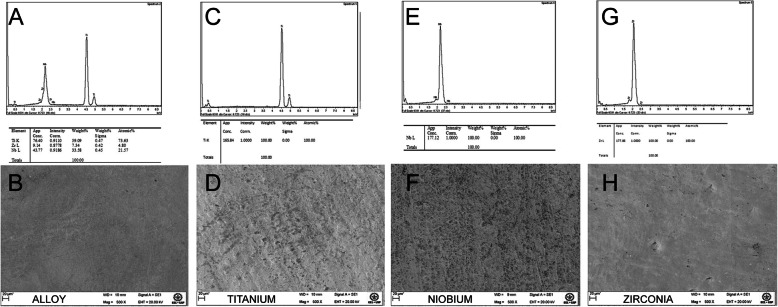
Energy-dispersive X-ray spectroscopy (EDS) showed the composition of the samples in percentage. **a** Titanium alloy—TiNbZr. **c** Pure titanium—Ti(cp). **e** Niobium—Nb. **g** Zirconia—Zr. Scanning electron micrograph images of the samples showed the differences of the surfaces. **b** Titanium alloy—TiNbZr. **d** Pure titanium—Ti(cp). **f** Niobium—Nb. **h** Zirconia—Zr

Energy-dispersive X-ray spectroscopy (EDS) gave the composition of the samples in percent of elements detected. The alloy presented a composition mainly of titanium, followed by the other compounds, niobium, and zirconia (Fig. 1a). Figures 1c, e, and g showed the samples with a high percentage of pure titanium, niobium, and zirconia, respectively. SEM images characterized and showed differences between the surfaces of the samples (Fig. 1b, d, f, h)

### Scanning electron microscope

**Fig. 2:**
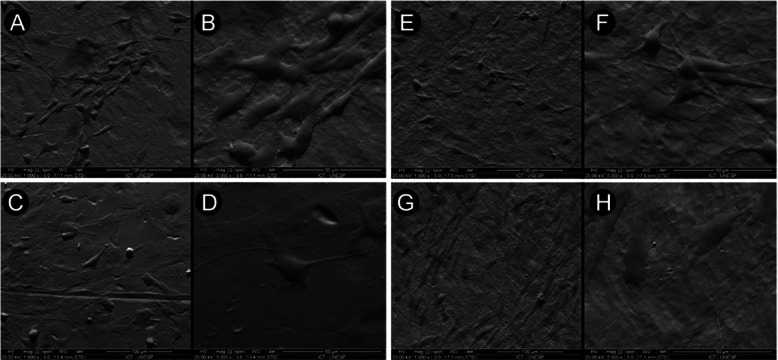
Scanning electron micrographs of the samples after cell culture showing cell adhesion and spread. At low magnification (**a**, **c**, **e**, **g**), it was possible to see cell adhesion. At high magnification (**b**, **f**, **d**, **h**), it was possible to see better cell interaction with the samples’ surfaces, with an increase of lamellipodia and filopodia, mainly in the titanium alloy and niobium samples. **a**, **b** TiNbZr; **c**, **d** pure titanium; **e**, **f** Nb; **g**, **h** Zr. Scale bar 50 μm

After 7 days of culture, the cell adhesion, morphology, and spreading on all the materials were observed through the SEM images, as demonstrated in Fig. 2. At low resolution (magnification × 1000), it was possible to see cell adhesion in all samples (Fig. 2a, c, e, g). At × 3000 magnification, it was possible to see the cells/surface interaction (Fig. 2b, d, f, h). At this magnification, increased lamellipodia and filopodia were observed on all surfaces, demonstrating the attachment and interaction between the cells and the surfaces.

### Cell viability

**Fig. 3:**
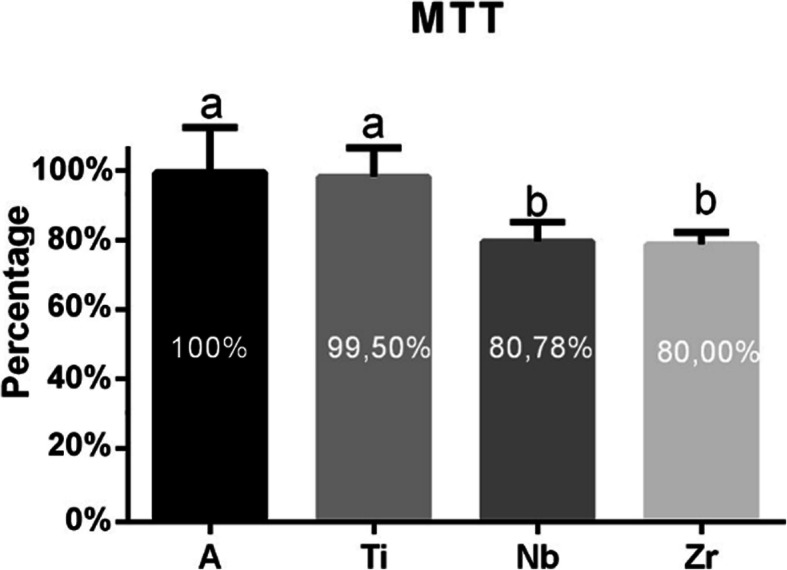
Graph showing (a) titanium alloy and Titanium cp with very similar results regarding cell viability and with higher cell viability when compared with the other samples. Statistical difference (a/b) were observed in relation to the groups of the basic elements (*p* < 0.05)

All groups presented cell viability above 70%, which was considered satisfactory according to ISO 9001/2000. It was possible to observe in the graphs shown in Fig. 3 that the pure titanium (control) and titanium alloy (A) sample presented the higher cell viability when compared with the other samples and were statistical different (*p* < 0.05).

### Cell proliferation

**Fig. 4:**
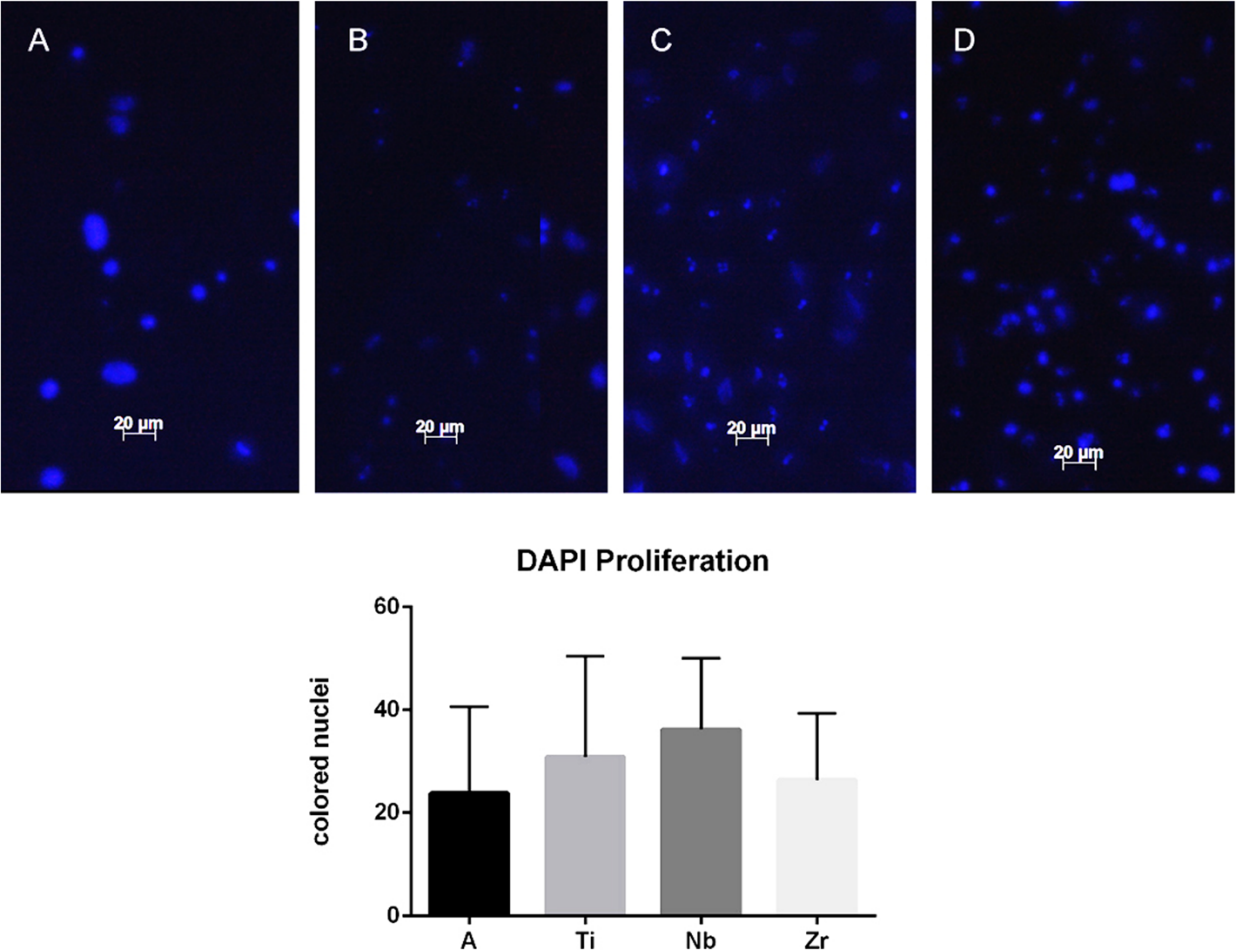
DAPI coloration was observed on all samples. The graph shows the Nb group (**c**) with higher cell proliferation, while the Zr group (**d**) exhibited the lowest number of stained nuclei. No statistical difference between the groups: **a** titanium alloy, **b** pure titanium, **c** niobium, and **d** zirconia

Cell proliferation was observed after 24 h of culture, showing stained nuclei. DAPI coloration was observed on all samples. After quantification, our results showed no statistical difference between the groups. Figure 4 shows cells on samples with blue nuclei, positive for DAPI coloration, and a graph demonstrating differences between the samples.

### Evaluation of osteogenesis

**Fig. 5:**
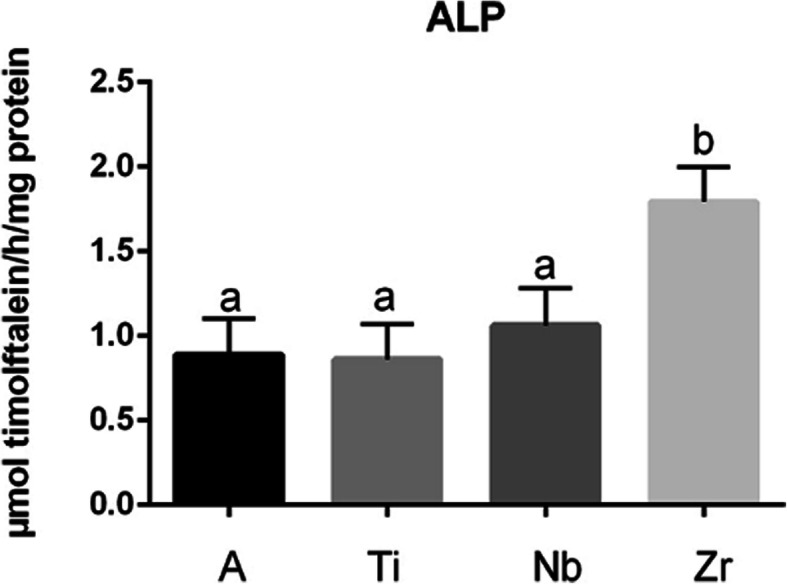
ALP graph showing high alkaline phosphatase activity for Zr group and statistically different (a/b) from all groups (*p* < 0.05)

**Fig. 6:**
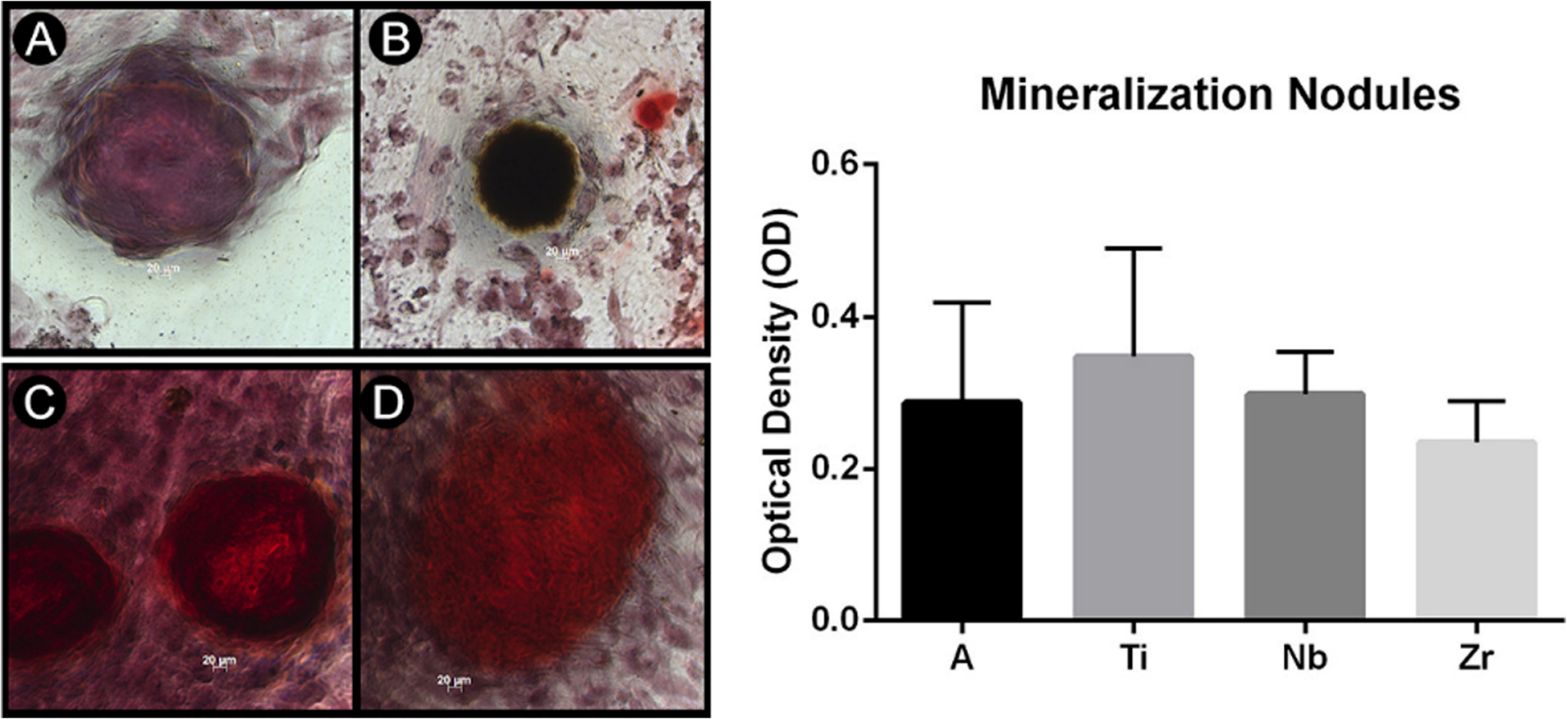
Mineralized eosinophilic nodules can be observed in all the groups. **a** Titanium alloy, **b** pure titanium, **c** Nb, and **d** Zr. The quantification of the mineralization nodules is shown by the graph. The Ti group presented the highest result and the Zr group the lowest. No statistical differences were found between groups

Figure 5 shows the Zr group expressing high alkaline phosphatase activity and was statistically different from all groups (*p* < 0.05). The Ti group, despite having the lowest result, did not present a statistical difference between groups A and Nb (Fig. 5). After 14 days of cell culture, mineralization nodules were observed in all the groups (Fig. 6). Figure 6 shows the quantification of the mineralization nodules. There is no statistical difference between the groups.

### Evaluation of the formation of microbial biofilms

**Fig. 7:**
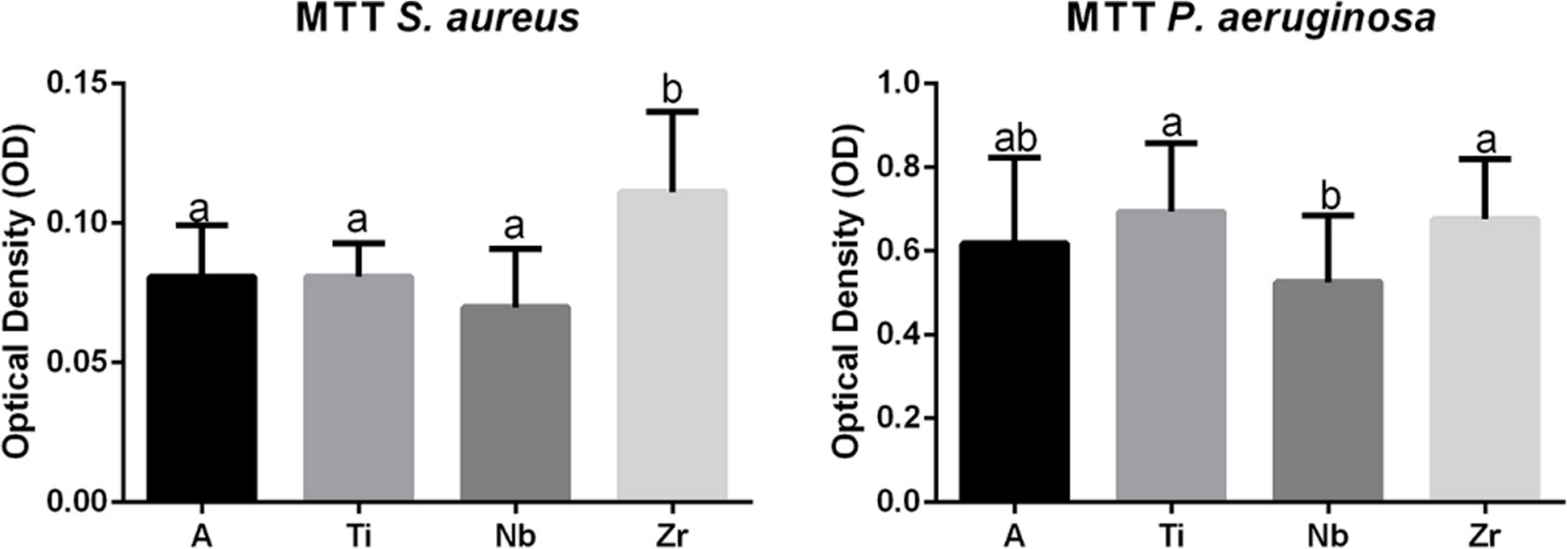
Nb group exhibited the lowest amount of *S. aureus* biofilm with statistical difference (a/b) from the Zr group, which presented the highest biofilm formation and differed statistically (a/b) from all groups (*p* < 0.05). For *P. aeruginosa* biofilm, the Nb presented the lowest biofilm values with statistical difference (a/b) from the Ti and Zr groups (*p* < 0.05). The Ti group showed the highest biofilm formation result, differing statistically (a/b) only from Nb

The MTT test measures the number of viable bacteria by the reduction reaction of the tetrazolium bromide (MTT). After 24 h of culture, the monotypic biofilms of *S. aureus* and *P. aeruginosa* were quantified by MTT and the optical density values represent bacterial viability. In the graph represented by Fig. 7, we observed that the Nb group exhibited the lowest amount of *S. aureus* biofilm, differing statistically only from the Zr, which presented the highest biofilm formation and differed statistically from all groups (*p* < 0.05). In the quantification of the *P. aeruginosa* biofilm, the Nb presented the lowest biofilm values again, differing statistically from the Ti and Zr groups (*p* < 0.05). On the other hand, the Ti group showed the highest biofilm formation result for *P. aeruginosa*, differing statistically only from Nb.

## Discussion

Currently, one of the most recurrent problems in the field of implantology is bacterial contamination, which can lead to implant loss. At the time of surgery for implant placement it is impossible to ensure an environment totally free of microorganisms, and the failure of dental implants can occur due to the adhesion of bacterial and biofilm formation disrupting or avoiding the bone integration process. As some studies have showed, the capacity of the host in developing a good inflammatory response and the characteristics of the biomaterial, including its composition, are directly involved in the successful process of wound healing and osseointegration [22–26].

Commercially, pure Ti and its alloys have been the main choice of biomaterials for bone implants, but mechanical and biological factors may impair their performance. Therefore, studies have been directed towards the production of new titanium alloys, with the addition of others metals, aiming to improve their characteristics, especially with respect to the modulus of elasticity and long-term cytotoxicity [27, 28]. Several studies have shown the effectiveness of metals such as Nb (niobium) and Zr (zirconia) being incorporated into titanium, resulting in a TiNbZr alloy that has some important characteristics (it is non-cytotoxic, has good biocompatibility, is lightweight, has a long life, and low elasticity modulus, which is important for preventing stress shielding) These characteristics make this biomaterial promising for bone replacement [29–34].

However, most studies with biomaterials and bone devices have not tested the ability of the metals individually in preventing bacterial contamination without compromising their capacity for cell proliferation and production of bone matrix. Jeyachandran et al. [35] affirm that the composition of biomaterials can modify surface charge states, which can influence their hydrophobicity and electrostatic interactions, resulting in bacterial adhesion. Therefore, our study was conducted to determine if each basic element (Ticp, Zr, and Nb) and alloy (TiNbZr) could influence bacterial biofilm formation and the behavior/response of bone marrow stromal cells in differentiating and producing bone matrix.

Adequate interactions between the biomaterial and bone environment are required and are initiated by cell adhesion on the surface of the implant, followed by proliferation and differentiation in osteoblasts for subsequent production of the mineralized extracellular matrix, thus promoting the fixation of the implant, a process called osseointegration [36]. The SEM images of our study showed all samples presented good adhesion and spreading cells. Mendonca et al. [37] affirm that adhesion is one of the most important steps in the osseointegration process and is related to the biocompatibility of the biomaterial. In the same way, Alselm et al. [38] believe that attachment, adhesion, and spreading of cells are the first phase of cell/material interactions; and that the quality of this interaction will influence the cells capacity to proliferate and to differentiate themselves in contact with the implant. Although we were able to observe cell interaction by SEM, we performed MTT to analyze the cell viability of the materials, and all samples showed good results, with better results for the TiNbZr alloy. Interestingly, only the metal Zr presented the smallest amount of MTT, cellular proliferation, and mineralized matrix in our study, contradicting some studies that reported good in vitro results with this element [39–42]. However, these studies used different cells and, according to Franco et al. [43], the cell activity may vary based on the type of cells used.

The choice of bone stromal cells and media without dexamethasone was proposed based on the use of these live cells therapeutically to increase bone repair and regeneration [44, 45] and trying to mimic the cellular environment in vivo, mainly because some studies have showed that use of this steroidal anti-inflammatory (Dex) in vitro induces osteoblast differentiation, but inhibits bone formation and induces bone resorption in vivo [20, 21]. The use of media without dexamethasone allowed us to analyze the osteoinductive potential of our biomaterials; which means the capacity of biomaterials in the recruitment of immature cells and the stimulation of these cells to develop into osteogenic cells [46]. The fact that bone marrow contains a heterogeneous population of cells, including mesenchymal cell lineages with the potential to become osteoblasts, and other osteoprogenitor cells at various levels of differentiation and maturity, made it one of the most viable options for use in bone regeneration [43].

Alkaline phosphatase (ALP) is one of the early proteins that regulate bone mineralization, and tests that evaluate alkaline phosphatase activity are considered a primary parameter indicative of cell differentiation into osteoblasts [47]. The results of our analysis of ALP activity allowed us to confirm that BMSC have the capacity to become osteoblasts without the use of dexamethasone. However, we cannot confirm if they have this capacity by themselves because, in a recent work, Kohno et al. [48] observed low ALP activity in these cells when comparing them with a population of mesenchymal stem cells isolated from bone marrow without the use of biomaterial as a promoter of cell differentiation. In the same way, Ghali et al. [49] tested BMSC to observe ALP expression by PCR, and the results showed no presence of gene expression. However, they were able to observe better production of mineralization nodules in media with dexamethasone 100 nm. They also observed that, in the media without dexamethasone, the cells were able to produce calcium in smaller amounts.

In our study, all biomaterials showed ALP activity, but Zr was the only sample that presented a statistical difference from the others groups. Matrix mineralization is the final event of bone formation [50]. In studies using osteogenic cultures, the quantification of mineralization is considered a functional parameter and reflects the advancement of cell differentiation [51]. The analysis of the cascade of events involved in bone matrix production should demonstrate a pattern of ALP activity followed by mineralization nodules on the same biomaterial. Our results with Alizarin Red dye showed an opposite pattern, and Ti presented more production of mineralization nodules, which are considered late-stage markers of bone formation. The results of the mineralization for Ti could indicate that metal has the potential to induce osteoblast maturation, and mature osteoblasts can produce bone matrix. However, no statistical difference was observed among samples. Our first hypothesis focused on the fact that Zr samples can induce more rapid osteoblastic differentiation of BMSC. This event is extremely important and desirable in the osseointegration process, as cells that become osteoblasts can produce proteins of bone matrix earlier, and faster osseointegration decreases the chances of bacteria contamination [52].

The success or failure of dental implants is directly related to the degree of integration of the implant material with the surrounding soft and hard tissues [53, 54] versus biofilm formation [55, 56]. Although Zr displayed better results for the ALP marker with respect to antimicrobial activity, this metal demonstrated the worst response for *S. aureus* with statistical differences. Zhao et al. [57] showed in their work that Zr can attract more biofilm compared to Ti and a TiZr alloy. Harris et al. [16] assert that *Staphylococcus aureus* was identified as an initial colonizer of dental implants and is the one bacterium which has the ability to attach to almost any type of titanium surface. In fact, we observed the presence of *S. aureus* on the samples with titanium. However, in our results, pure titanium showed a high affinity to *P. aeruginosa* which agreed with the results of Truong et al. [14]. Albertini et al. [12] showed in an in vivo study that *P. arginosa* and *S. aureus can* be observed on titanium surfaces, and they believe that these microorganisms may be associated with implant failure. Some studies have shown TiNbZr as a good option due to its excellent mechanical, bioactivity, and corrosion protection [58, 59]. Compared with pure titanium and zirconia, this alloy had more potential to avoid *P. arginosa* and *S. aureus*. However, Nb was selected in our study as the best material to prevent bacteria contamination, since it showed low affinity for both biofilms and, therefore, we believe it has antimicrobial proprieties.

## Conclusion

Our study allowed us to conclude that none of the metals alone (Ti, Nb, Zr) or the alloy (Ti-35Nb-7Zr) has all the properties individually to avoid microbial biofilm formation and induce cell differentiation. However, we confirmed that all samples and titanium alloys have osteoinductive potential and can induce BMSC differentiation. We also determined that Zr has the best potential for this purpose. It was suggested that Nb could also contribute to the avoidance of bacterial formation and we believe that, when it is incorporated into an alloy, it can help prevent bacterial contamination. Furthermore, the use of dexamethasone should be further analyzed as an inductor of cell differentiation in research using biomaterials, because the use of this drug can cover up the osteoinductive proprieties of the biomaterials. The use of BMSC from mice femurs, in our view, proved the potential of these cells to differentiate into osteoprogenitor cells, making them a possible source of bone tissue therapy.

## Data Availability

Not applicable.
